# Croatian Native Grapevine Varieties’ VOCs Responses upon *Plasmopara viticola* Inoculation

**DOI:** 10.3390/plants12020404

**Published:** 2023-01-15

**Authors:** Petra Štambuk, Iva Šikuten, Darko Preiner, Edi Maletić, Jasminka Karoglan Kontić, Ivana Tomaz

**Affiliations:** 1 Department of Viticulture and Enology, Faculty of Agriculture, University of Zagreb, Svetošimunska Cesta 25, 10000 Zagreb, Croatia; 2 Centre of Excellence for Biodiversity and Molecular Plant Breeding, Svetošimunska Cesta 25, 10000 Zagreb, Croatia

**Keywords:** *Vitis vinifera* L., downy mildew, secondary metabolites, SPME-*Arrow*-GC/MS

## Abstract

The *Plasmopara viticola* pathogen causes one of the most severe grapevine diseases, namely downy mildew. The response to *P. viticola* involves both visible symptoms and intricate metabolomic alterations, particularly in relation to volatile organic compounds, and depends on the degree of resistance of a particular variety. There are numerous native grapevine varieties in Croatia, and they vary in susceptibility to this oomycete. As previously reported, in vitro leaf disc bioassay and polyphenolic compound analysis are complementary methods that can be used to separate native varieties into various resistance classes. This research used the Solid Phase Microextraction-Arrow Gas Chromatography-Mass Spectrometry method to identify the early alterations in the VOCs in the leaves after *P. viticola* inoculation. Based on the absolute peak area of sesquiterpenes, some discrepancies between the sampling terms were noticed. The presence of certain chemical compounds such as humulene, ylangene, and *α*-farnesene helped distinguish the non-inoculated and inoculated samples. Although specific VOC responses to *P. viticola* infection of native varieties from various resistance classes could not be identified, the response of less susceptible native varieties and resistant controls was associated with an increase in the absolute peak area of several compounds, including geranylacetone, *ß*-ocimene, and (*E*)-2-hexen-1-ol.

## 1. Introduction

In addition to insects and weeds, pathogenic microorganisms cause structural and/or functional damage to plants, resulting in biotic stress. Plant-pathogen interactions can be viewed as a two-way communication process in which not only is the plant able to recognise a foreign organism and defend itself against it, but the pathogen must also be able to manipulate the plant’s biology to create an optimal environment for its own growth and development while avoiding the plant’s response [1]. The first line of plant defence can be triggered through the pathogen-associated molecular pattern (PAMP) system, which recognizes the pathogen. This is followed by a series of signal changes that finally impart a defence strategy, also known as the “zigzag” model [2]. Photosynthesis-related alterations, pathogenesis-related protein synthesis, restructuring of the cytoskeleton, generation of reactive oxygen species, and activation of programmed cell death constitute the fundamental level of plant pathogen recognition modifications [3]. Due to the plasticity of the plants in response to the pathogen and the establishment of either a compatible or incompatible interaction, the modulation of several classes of primary and secondary metabolites alters. During the initial phases of the infection, plant reactions to oomycetes are identical, albeit less effective and slower in susceptible plants [4].

Unlike primary metabolites, such as carbohydrates, lipids, and proteins, which are directly involved in plant development and growth, secondary metabolites are multifunctional compounds that are typically involved in the plant’s defence system or they act indirectly, mediating the signals between different parts of the same plant, from plant to plant and between plants and other organisms. Due to their sessile nature, plants synthesize these compounds to repel herbivores, build barriers against pathogen invasion and mitigate oxidative stress [5]. On the basis of the accumulation and concentration of secondary metabolites in grapevine berries, such as polyphenolic and volatile organic compounds, it has been discovered that it is possible to classify *V. vinifera* varieties into genetic-geographic groups [6,7].

Terpenes, alkanes, alkenes, alcohols, esters, and acids belong to the class of volatile organic compounds. Terpenes are the largest and most researched class of these compounds. Their building block is a five-carbon isoprene unit. Through the condensation of two or more isoprene units, mono-(C_10_), sesqui-(C_15_), and diterpene (C_20_) precursors are formed [8]. VOCs perform essential functions in how plants interact with other organisms and how they respond to biotic stress. They constitute about 1% of the secondary metabolites found in plants. Due to their low molecular weight and high vapour pressure, these lipophilic molecules freely diffuse into the environment and pass through biological membranes. Typically, pathogen-induced volatile organic compounds (VOCs) are composed of methyl salicylate (MeSA), mono- and sesquiterpenes, heterocyclic compounds, green leaf volatiles (GLVs), and ketones [9,10,11]. Plants continuously produce GLVs, such as C_6_ aldehydes, alcohols, and esters, and do so to a greater extent under stress conditions [12]. 

In grapevine, both the defensive role and accumulation of volatile organic compounds, following an attack by *Plasmopara viticola* [(Berk. et Curt.) Berl. et de Toni], have been demonstrated. Some volatile organic compounds, such as 2-phenylethanol, 2-ethylfuran, (*E*)-2-pentenal, *ß*-cyclocitral, *ß*-caryophyllene, and *ß*-selinene, inhibited *P. viticola* infection in leaf tissues. The abundance of the studied VOCs was greater in resistant (BC4, Kober 5BB, SO4, and Solaris) genotypes than in susceptible genotypes (Pinot noir) [11]. Monoterpenes and sesquiterpenes are found in higher quantities in SO4 and Kober 5BB plants compared to Pinot noir plants after inoculation [13]. The direct antibacterial action of four selected compounds (farnesene, nerolidol, ocimene, and valencene) as well as the role of leaf terpenes in the resistance mechanism of two resistant cultivars (Mgaloblishvili, a pure *V. vinifera* cultivar, and Bianca, an interspecific hybrid) were determined [8]. Benzaldehyde has been suggested as a putative biomarker of resistance because it acts as a stimulator of salicylic acid (SA)-mediated defence [14]. It was also discovered in greater amounts in mono-locus (Solaris) and pyramided (F12P60) resistant genotypes. Farnesene was abundantly expressed in three mono-locus resistant genotypes (BC4, Bianca, F12P160). Linalool was substantially more abundant in Bianca, whereas (*E*)-nerolidol and neral distinguished the pyramidal genotype F12P60 [15].

*P. viticola*, the causal agent of grapevine downy mildew, is an obligate biotrophic oomycete native to North America. Therefore, European grapevine (*V. vinifera*) varieties are generally susceptible to this pathogen since it was introduced in this area in the late 19th century [16]. Biotrophic microorganisms have an important function in the ecosystem because they decompose organic matter [17]. However, when *P. viticola* sporangia develop in large quantities under favourable weather conditions (temperatures between 20 and 25 °C and leaf wetness) [18], they cause disastrous consequences such as defoliation, reduced and/or complete loss of grape quality and quantity [16,19]. In conventional vineyards, downy mildew epidemics cause severe economic losses when fungicides are not administered. Due to the detrimental effects of chemical pesticides on the environment, and human and animal health, as well as the emergence of pathogens resistant to these treatments [20,21,22], the European Union has restricted their usage through the Farm to Fork Strategy [23]. Today, the focus lies on the development of alternative tools, such as the breeding of resistant cultivars, the development of new active substances, and the search for natural compounds and biocontrol agents that can be applied individually or in combination to eradicate the pathogen or mitigate its effects [4]. 

The genetic diversity of traditional grapevine varieties is an inexhaustible source of traits potentially useful in the upcoming challenging environmental conditions. Therefore, they should not be neglected since each variety has a unique characteristic that differentiates it from all other varieties. There is a possibility that these characteristics will become desirable or valuable in the future, despite the fact that they may seem mundane at present. Their preservation and continuous research are of utmost importance for maintaining biodiversity and expanding the scope of future breeding programs [24]. Quantitative trait loci (QTL) named *Rpv*, an acronym for resistance to *P. viticola*, are responsible for grapevine’s resistance response. The Table of Loci for Traits in Grapevine Relevant for Breeding and Genetics (https://www.vivc.de/, the access date: 15 November 2022) lists and describes the 31 QTLs discovered in *Vitis* species to date. Most of them have been found in the *Muscadinia* subgenus, along with several wild North American and Asian *Vitis* species. However, the last three QTLs named *Rpv29*, *Rpv30*, and *Rpv31*, have been identified in the Georgian *V. vinifera* variety of Mgaloblishvili [25] which confirms the importance of preserving and studying rare varieties that are cultivated in limited areas.

The 4th century BCE saw the beginning of viticulture production in Croatia, both in the continental Pannonia region with the arrival of Celts and along the eastern Adriatic coast where Greeks founded cities [26]. Due to turbulent historical events and the introduction of mildews (*P. viticola* and *Erysiphe necator*) and phylloxera (*Daktulosphaera vitifoliae*) from the North American continent during the 19th century, the number of grapevine varieties and vineyard areas have changed significantly since then. Consequently, today’s Croatian grapevine biodiversity consists of about one hundred varieties [27]. According to previous research conducted on some of these varieties, their susceptibility to the downy mildew disease under field conditions varies. Moreover, this was confirmed in controlled laboratory conditions using the leaf disc bioassay according to the OIV 452-1 descriptor [Leaf: degree of resistance to *Plasmopara* (leaf disc test)]. After measuring chlorophyll fluorescence and multispectral imaging traits, it is possible to distinguish non-infected and infected leaf discs 24 h after inoculation, whereas, on the fourth day upon inoculation, the differences between varieties belonging to various OIV classes are observed [28]. As far as secondary metabolites are considered, it was found that the constitutive polyphenolic profile of leaves is responsible for genotype differentiation among the OIV classes of resistance to *P. viticola* [29].

This study’s primary objective was to expand the analysis of secondary metabolites in leaves before and during the early stage of inoculation, with a particular emphasis on volatile organic compounds. The research was performed using Solid Phase Microextraction (SPME)–Arrow Gas Chromatography–Mass Spectrometry (GC–MS). Similar methods have been used previously [8,11,14,15], but this one has proven to be particularly effective for the detection of VOCs [30]. Defining the differences between the metabolomic profiles of susceptible and resistant grapevines and detecting the resistance–related metabolites could broaden and introduce new concepts in plant protection strategies. Moreover, the detection of VOC emission patterns could be used to screen hybrids with varying resistance levels or to diagnose diseased plants [31].

## 2. Results

Eighty-six VOCs were identified through SPME-*Arrow*-GC/MS analysis of 17 genotype leaf samples collected at 0, 24, 48, and 94 h after *P. viticola* inoculation. There were 19 alcohols, 18 carbonyls (aldehydes and ketones), 17 monoterpenes, 10 sesquiterpenes, 10 esters, 9 acids, and 3 compounds that belong to some other groups of compounds, such as C_13_-norisoprenoids and lactones, were detected (Tables S2 and S3). As far as individual compounds are concerned, the highest absolute peak areas were detected for 2-hexenal, benzyl alcohol, 3-hexen-1-ol, nonanal, and 4-hexen-1-ol acetate that were calculated as average values of all analysed samples. The interactions of all three factors (sampling term, treatment, and OIV resistance class), including T_0,_ were significant for 17 identified VOCs, the majority of which were carbonyls (5), alcohols (4) and acids (3). The mean values of the interactions are presented in Table S2.

### 2.1. Changes throughout the Terms of Sampling

Of the 86 identified VOCs, 49 compounds significantly contributed to the ability to distinguish between the terms of sampling upon inoculation regardless of treatment and OIV class of resistance (Table S3). A slightly higher number of compounds increased significantly upon inoculation in at least one term (28) compared to the number of compounds that decreased (18). The most numerous increasing compounds were those that belong to monoterpenes (7), alcohols (6), and sesquiterpenes (5).

The alcohols with the highest APA in T_1_ were: 1-nonanol, 1-butoxy-2-propanol, 1-methoxy-2-propanol, and *α,α*-dimethylbenzyl alcohol, whereas the alcohols with the lowest APA in T_3_ were 3,7-dimethyl-1,7-octanediol and benzyl alcohol. The ascending significant change between all three terms of sampling was obtained for 1-hexanol, (*E*)-2-hexen-1-ol, 2-ethyl-2-hexen-1-ol, and 3-hexen-1-ol. However, the 2-ethyl-1-hexanol APA plunged significantly from T_1_ to T_3_. Significant differences between T_1_ and T_3_ were observed for 1-octen-3-ol with higher APA in T_1_, whereas phenylethyl alcohol was more abundant in T_3_.

In T_1_, the 2-hexenal APA was significantly the lowest, whereas the (*E*)-2-nonenal, acetophenone, nonanal, and octanal APAs were the highest. The APA of benzaldehyde decreased significantly throughout the terms following the inoculation, whereas a significant increment was observed for 6-methyl-5-hepten-2-one from T_1_ to T_3_. The APA of (*E,Z*)-2,6-nonadienal and (*E,E*)-3,5-octadien-2-one distinguished between T_1_ and T_3_ with the highest APA being observed in T_3_ and T_1_, respectively. The APA of hexanal did not change significantly in the first two terms following the inoculation, although it increased significantly in the third term.

Markedly, the APA of (*E*)-3-hexenyl butanoate and (*Z*)-2-hexenyl acetate was lowest in T_3_. The lowest APA of hexyl acetate, phenylmethyl formate, and methyl salicylate was observed in T_1_. A higher APA of ethyl octanoate in T_1_ distinguished it significantly from T_3_.

In terms of monoterpenes, citronellol, neral, and *α*-terpineol had significantly lower APAs in T_1_, whereas *p*-cymene had a significantly lower APA in T_3_. 

Significantly, the highest APAs in T_1_ and T_2_ were observed for geranyl vinyl ether and (*E*)-linalool oxide, respectively. The APAs of (*Z*)-linalool oxide and *ß*-ocimene increased significantly from T_1_ to T_2_, whereas geranylacetone increased significantly from T_1_ to T_3_.

The *α*-farnesene sesquiterpene increased significantly from T_1_ to T_3_, whereas from T_1_ to T_2_ the same is true for humulene and *ß*-guaiene. Finally, compounds belonging to another group of VOCs, such as (*E*)*-ß*-ionone, dihydroactinidiolide, and 5-ethyl-2(5H)-furanone, also increased from T_1_ to T_2_.

### 2.2. Differences between Non-Inoculated and Inoculated Leaves

Compounds that belong to sesquiterpenes and alcohols contributed the most to the discrimination between non-inoculated and inoculated leaves, regardless of the sampling term following the inoculation and the OIV resistance class. More precisely, the infected samples contained significantly elevated levels of humulene, ylangene, and *α*-farnesene, as well as 1-hexanol, (*E*)-2-hexen-1-ol, and 2,4-dimethyl-3-pentanol. In contrast, non-infected samples contained significantly higher APAs of 2-ethyl-1-hexanol, 1-nonanol, and 1-butoxy-2-propanol alcohols.

Among the remaining compounds, inoculated leaves measured significantly higher monoterpene geranylacetone, aldehyde octanal, ketone 6-methyl-5-hepten-2-one, and 2-hexonoic acid APAs (Table S3).

### 2.3. Differences between the OIV Resistance Classes

As previously explained, each genotype included in this research was assigned to the appropriate OIV class of resistance based on the severity of *P. viticola* sporulation and the OIV 452-1 descriptor. Although only seven out of eighty-six identified compounds failed to significantly differentiate the OIV resistance classes, no clear separation of genotypes into the OIV resistance classes was obtained. Thus, the contribution of individual compounds is described below.

The lowest APA of 2-hexenoic acid distinguished the completely resistant OIV class 9 from all other OIV classes, whereas the low APA of pentanoic acid distinguished OIV classes 1 and 9 from classes 3, 5, and 7. The lowest APAs of (*E*)-3-hexenoic acid and decanoic acid were observed in OIV class 3 compared to other OIV classes. Benzoic acid was significantly the most abundant in OIV class 5, whereas heptanoic acid was the most abundant in OIV classes 1 and 5. Decanoic acid distinguished OIV class 3 by its low APA compared to other classes.

The highest APA of 2-ethyl-1-hexanol distinguished the most susceptible OIV class 1 from the highly resistant OIV class 7, and the resistant OIV class 9. Similarly, the APA of 1-nonanol distinguished OIV class 1 and OIV class 9, in which the lowest APA of this alcohol was observed. OIV class 3 had the lowest APA of 1-octanol. Alcohols 2-ethyl-2-hexen-1-ol and (*E*)-2-hexen-1-ol were found in the highest APA in OIV classes 7 and 5, respectively. OIV classes 3 and 9 were specified by the lowest APA of 3-hexen-1-ol. The highest APAs of 3,7-dimethyl-3-octanol, 2,4-dimethyl-3-pentanol, benzyl alcohol, and phenylethyl alcohol distinguished OIV class 9 from all other classes.

As far as carbonyls are concerned, a higher APA of (*E,E*)-2,4-heptadienal differentiated OIV classes 1, 3, and 5 (pure *V. vinifera* varieties) from OIV classes 7 (interspecific hybrid Solaris) and 9 (*V. riparia*). Similarly, (*E,Z*)-2,6-nonadienal was observed in significantly higher APA in pure *V. vinifera* varieties compared to *V. riparia*. Aldehydes 2-hexenal and (*E*)-2-nonenal and their higher APAs discriminated OIV classes 1 and 3 from other classes, whereas a higher APA of (*E,E*)-3,5-octadien-2-one distinguished the susceptible OIV classes 1 and 3 from the partially resistant and resistant OIV classes 5, 7 and 9. OIV class 9 was specified by the highest APA of 4-pentenal compared to all other classes. The APA of 6-methyl-5-hepten-2-one was the highest in OIV class 7 followed by OIV class 9 and then OIV classes 5 and 1. Finally, the lowest APA of this ketone separated OIV class 3 from others. The highest APA of 2,5-dimethyl-benzaldehyde and heptanal distinguished OIV classes 9 and 5 from others, respectively. A higher APA of hexanal separated OIV class 5 from classes 1 and 3. Nonanal and octanal were highest in OIV classes 5 and 7 and lowest in OIV class 3.

The APA of phenylmethyl acetate was the highest in OIV class 9, followed by OIV classes 1 and 5, and the lowest in OIV classes 3 and 7. Significantly the highest APA of ethyl octanoate discriminated OIV class 1 from others. Similarly, 3-hexenyl butanoate distinguished OIV classes 1 and 5 from others. Significant differences were observed for (*E*)-2-hexenyl benzoate and its higher APA in OIV classes 3 and 5 compared to OIV class 7. In contrast, phenylmethyl formate had a higher APA in OIV class 7 compared to classes 1, 3, and 5. The highest APA of ethyl octanoate distinguished OIV class 1 from others, whereas the lowest APA of (*Z*)-2-hexenyl acetate was obtained in OIV class 9.

Among monoterpenes, the APA of *ß*-cyclocitral did not distinguish OIV classes 7 and 9, although it separated OIV class 9 from classes 1, 3, and 5 by being less abundant in OIV class 9. The highest APA of citronellol and nerol differentiated OIV class 7 from others. OIV class 9 was specific by the highest APA of (*Z*)-linalool oxide, eucalyptol, and *p*-cymene. A higher APA of geranylacetone differentiated OIV classes 7 and 9 from others. The highest APA of limonene was obtained in the most susceptible OIV class 1. In the same way, menthol distinguished OIV class 1 from classes 7 and 9, whereas linalool separated OIV class 1 from classes 3, 5, and 9. Low APA of *α*-terpineol distinguished OIV classes 3 and 5 from others. Higher APA of *ß*-ocimene differentiated OIV classes 1 and 3 from class 5. Geranylacetone was the most abundant in OIV class 9 followed by OIV class 7, whereas it did not distinguish OIV classes 1 and 5.

Higher APAs of (*Z*)*-ß*-farnesene and *α*-farnesene differentiated OIV classes 7 and 9 from others. In comparison, the APAs of humulene and *ß*-guaiene were the highest in OIV classes 1 and 5. OIV class 9 was specific due to the lowest APA of 5-ethyl-2(5H)-furanone, whereas a higher APA of dihydroactinidiolide was detected in OIV class 5 compared to OIV classes 1 and 3 (Table S3).

### 2.4. Compounds Related to Higher Resistance

Aiming to identify VOCs that could be responsible for higher resistance to *P. viticola*, highly positive correlations between the infected samples and genotypes that belong to OIV classes 5, 7, and 9 were sought throughout the experiment. At the same time, negative or low correlations were sought for the non-infected (control) samples to verify that the ascending APA of a VOC in the infected sample is associated with a defence mechanism against infection. Moreover, when the same trend was observed for VOCs in varieties that are most susceptible to *P. viticola* (OIV class 1), these compounds were excluded from consideration.

Throughout the experiment, highly positive correlations were obtained for 2-hexenoic acid in samples from all three OIV class 5 (Malvazija istarska, Ranfol, and Teran) infected varieties, whereas negative or low correlations were obtained for non-infected (control) samples of the same resistance class. The same is true for the following VOCs in Malvazija istarska specifically: 2-ethyl-2-hexen-1-ol, *α,α*-dimethylbenzyl alcohol, phenylmethyl acetate, phenylmethyl formate, limonene, *α*-terpineol, *ß*-myrcene, *ß*-ocimene, geranylactone, copaene, *α*-farnesene, and *γ*-muurolene. The infected samples of Teran exhibited highly positive correlation for (*E*)-2-hexen 1-ol, (*E*)-2-hexenyl benzoate, *ß*-cyclocitral, citronellol, linalool, and *p*-cymene, whereas Malvazija istarska and Ranfol exhibited the same for benzeneacetaldehyde, ethyl benzoate, citronellol, menthol, caryophyllene, humulene, and *α*-muurolene. However, the same trend was observed for the majority of the above-mentioned compounds in varieties that are the most susceptible to *P. viticola* (OIV class 1) (Table S4). Therefore, they cannot be considered the cause of resistance reactions.

**Figure 1:**
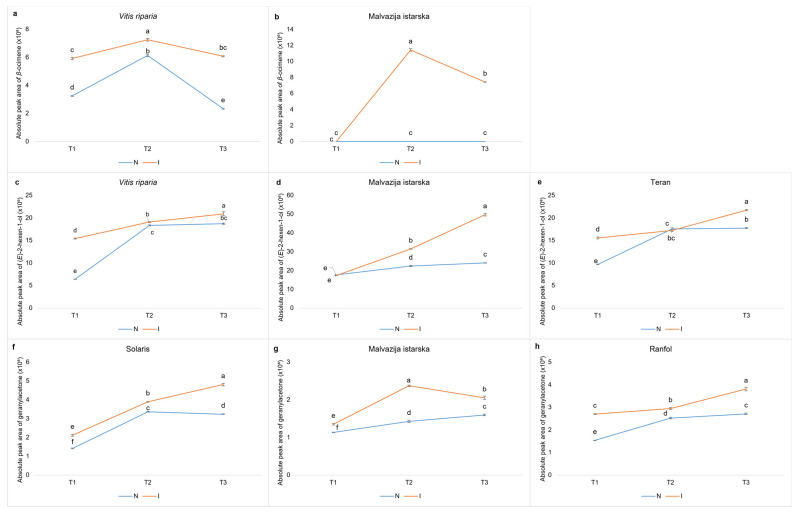
The absolute peak areas of *β*-ocimene, (*E*)-2-hexen-1-ol, and geranylacetone in the sampling terms upon *P. viticola* inoculation (24 hpi (T1), 48 hpi (T2) and 96 hpi (T3)) for inoculated (I) and non-inoculated (N) samples of genotypes that belong to resistance classes 5 (Malvazija istrska, Ranfol, and Teran), 7 (Solaris), or 9 (*Vitis riparia*). The differences between the means were evaluated by Duncan’s multiple range test at a confidence level of 95% (*p* < 0.05). Different letters indicate statistical significance. Sub-figures depict the absolute peak areas of volatile organic compounds as follows: (**a**) *β*-ocimene in *Vitis riparia,* (**b**) *β*-ocimene in Malvazija istarska, (**c**) (*E*)-2-hexen-1-ol in *Vitis riparia*, (**d**) (*E*)-2-hexen-1-ol in Malvazija istarska, (**e**) (*E*)-2-hexen-1-ol in Teran, (**f**) geranylacetone in Solaris, (**g**) geranylacetone in Malvazija istarska, and (**h**) geranylacetone in Ranfol.

Nevertheless, a few compounds detected in OIV 5 were also detected in OIV 7 or 9 but not in OIV 1, having a high positive correlation in infected samples and a low or negative correlation in non-infected ones. In particular, VOCs detected in OIV 9 were ocimene, (*E*)-2-hexen-1-ol, whereas geranylacetone was found in OIV 7. Figure 1 depicts the APA changes of specific VOCs in response to inoculation, which may have contributed to the increased resistance of genotypes belonging to OIV classes 5, 7, or 9.

### 2.5. Sesquiterpenes and the OIV Resistance Classes

**Figure 2:**
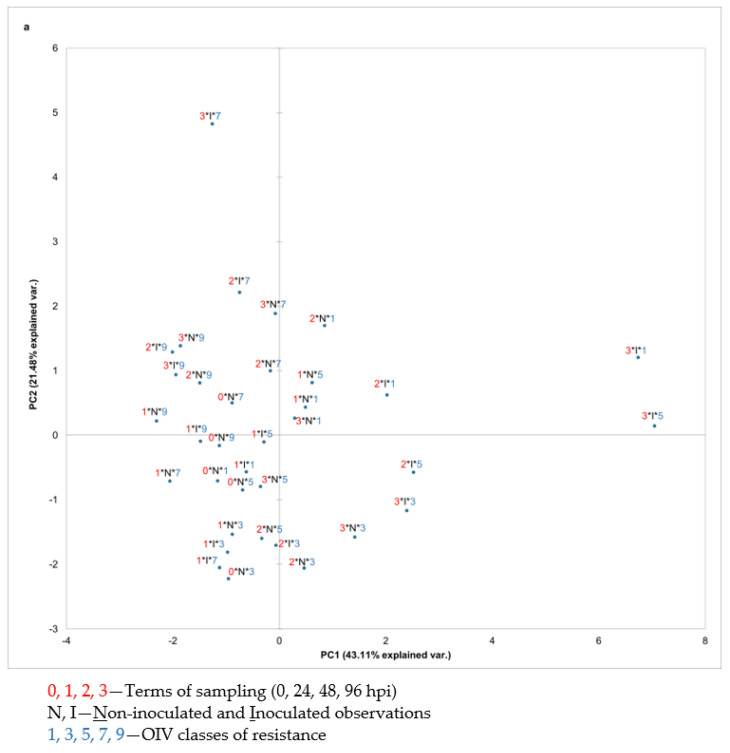

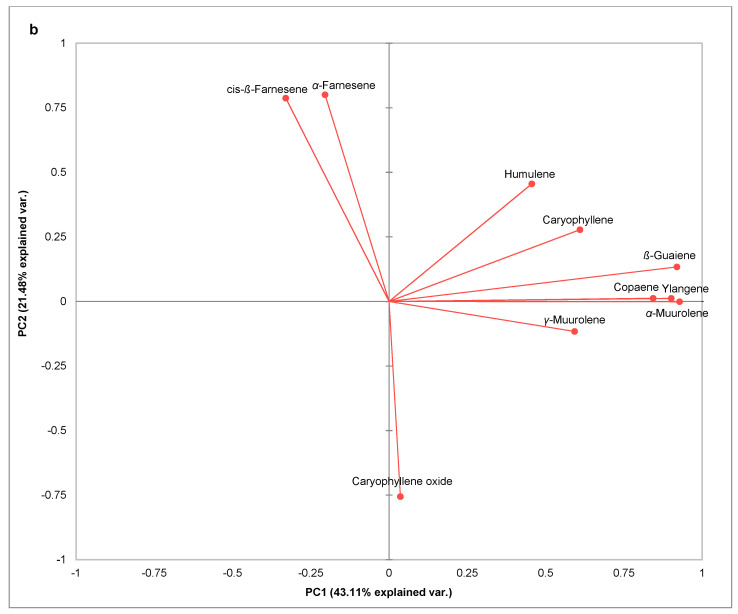
PCA scatter plot depicting (**a**) OIV classes of resistance (1, 3, 5, 7, and 9 from the most susceptible to totally resistant) based on their leaves’ sesquiterpenes absolute peak area before and after artificial *P. viticola* inoculation at 0, 24, 48 and 96 hpi in the space defined by the first two principal components explaining 64.59% of the variability; (**b**) the vector diagram of correlation among the absolute peak area of sesquiterpenes and the first two principal components.

To analyse the total variability of the volatile compounds’ absolute peak area related to the division of the OIV resistance classes (1, 3, 5, 7, and 9), terms of sampling (0, 24, 48, and 96 hpi), and treatments (considering non-inoculated and inoculated samples), a principal component analysis (PCA) was performed using all detected VOCs and using each group of detected volatile compounds separately. Most groups of compounds did not contribute to a distinct separation of the samples by any of the aforementioned factors (data not shown). However, the PCA based on the APA of individual sesquiterpenes contributed the most to distinguishing the OIV classes of resistance. In particular, the PCA scatter plot of the first two components explained 64.59% of the variability (Figure 2) with the first principal component (PC1) accounting for 43.11% and the second (PC2) for 21.48%. The projection on these two axes distinguished the two highly resistant genotypes (OIV classes 7 and 9) from *V. vinifera* genotypes (OIV classes 1, 3, and 5). However, in OIV classes 1, 3, and 5, terms and treatments were not clearly separated (Figure 2a).

Based on the related vector diagram (Figure 2b), it is possible to define the sesquiterpenes that contributed to such distribution and grouping of samples that belong to either OIV classes 7 and 9 or OIV classes 1, 3, and 5 in the space defined by the first two principal components. One group containing all the samples belonging to OIV classes 7 and 9 regardless of the treatment and sampling term was separated from the other group due to the higher APA of *α*-farnesene and (*Z)-ß*-farnesene. Most of these observations are located in the second quadrant and a few of them are in the third quadrant.

## 3. Discussion

*P. viticola* maintains its life cycle in living tissue as its tubular mycelium grows intercellularly and obtains nutrients by parasitizing the host cells through haustoria [32]. Leaves are typically the first to show symptoms of downy mildew, especially young leaves that have not yet developed ontogenic resistance to the disease [33]. Biosynthesis of VOCs occurs in the leaf mesophyll tissues, specifically in the palisade mesophyll cells [34]. Consequently, since phytopathogens alter plant VOCs emission, decreasing or increasing the amount of some pre-existing VOCs, and inducing the appearance of newly synthesized VOCs, this research was conducted on young leaves and their volatile organic compounds (VOCs) [35]. It is known that VOCs can directly inhibit pathogen growth, induce plant resistance mechanisms in neighbouring plants, and mediate associational resistance by adsorption to the cuticle of receiver tissues [36]. During the early stages of infection, the susceptible cultivar undergoes the following changes: at 24 hpi, the zoospores germinate and the germ tube penetrates the substomatal cavity; at 48 hpi, the *P. viticola* hyphae are observed in the intercellular spaces; at 96 hpi, the sporangiophores begin to develop from the stomata [37]. A novel SPME-Arrow GC/MS technique proved to be efficient for this kind of analysis by processing a large number of samples and providing a whole range of VOCs [30].

As mentioned previously, phenotypic differences among Croatian native varieties have been investigated, and some specificities corresponding to OIV resistance classes have been observed. Based on the content and composition of polyphenolic compounds, it was found that their constitutive profiles in leaves are responsible for diverse levels of resistance to *P. viticola* [29]. The analysis of 86 volatile organic compounds (VOCs) in the leaves of 14 native Croatian varieties, including Chardonnay, Solaris, and *V. riparia*, was performed with the intention of expanding the current findings based on secondary metabolites. To the best of our knowledge, this is the first time such extensive research on this topic has been conducted. Although a clear separation of differently resistant genotypes was not accomplished, as the inoculation time progressed, some specificities were defined among the OIV classes of resistance and the APA of sesquiterpenes. Moreover, a few compounds, such as geranylacetone, *ß*-ocimene, and (*E*)-2-hexen-1-ol could be responsible for a higher resistance of OIV classes 5, 7, and 9.

Considering the sampling terms upon *P. viticola* inoculation, a slightly higher number of VOCs was detected in increased APAs over time, based on the average values of all 17 analysed genotypes. This has already been observed for the resistant genotypes of BC4, Kober 5BB, SO4, and Solaris whose leaf VOCs were analysed at 6 dpi and compared to 0 dpi, whereas the APA of VOCs in Pinot noir leaves decreased [11]. Although benzaldehyde was not an indicator of *P. viticola* inoculation, its APA decreased throughout the experiment which is in accordance with Ricciardi et al., (2021) [8] whose samples were frozen likewise. At the same time, benzaldehyde content increased in the Bianca cultivar when fresh leaves were analysed [14], meaning that VOC emission patterns could be related to the sample preparation and the term of sampling. The most numerous increasing compounds were alcohols (e.g., 3-hexen-1-ol and 2-ethyl-2-hexen-1-ol), monoterpenes (e.g., citronellol and neral), and carbonyls (e.g., hexanal and (*E,Z*)-2,6-nonadienal). These groups of VOCs increased in the leaves of Bianca and Mgaloblishvili [8].

Terpenes were found to be the most discriminative among the OIV classes of resistance. The high APA of sesquiterpenes *α*-farnesene and (*Z)-ß*-farnesene distinguished OIV classes 7 and 9 from the more susceptible OIV classes 1, 3, and 5 regardless of the treatment and the sampling term. Moreover, *α*-farnesene was suitable for distinguishing treatments throughout the experiment due to its higher APA in inoculated leaves compared to non-inoculated ones. Similarly, increased emission of sesquiterpenes were detected in in vitro plantlets of the downy mildew–resistant genotypes of SO4 and Kober 5BB, whereas the increment of these VOCs was lower in the Pinot noir susceptible variety [13]. Likewise, an increased amount of farnesene was detected in the resistant genotypes of Mgaloblishvili and Bianca upon *P. viticola* inoculation together with the up-regulation terpene synthase genes, suggesting a pathogen-dependent transcriptional regulation of terpene biosynthesis [8]. In a study conducted by Ciubotaru et al., (2021), farnesene was expressed in high concentrations in the genotypes with mono-locus resistance, namely BC4 (*Rpv1*), Bianca (*Rpv3-1*) and F12P160 (*Rpv12*), and in the pyramided resistant genotype F12P127 (*Rpv3-1*, *Rpv3-3*, *Rpv10*) [15]. These findings indicate that VOCs, especially sesquiterpenes, produced by downy mildew-resistant genotypes contribute to grapevine defence against *P. viticola*. 

Algarra Alarcon et al., (2015) [13] detected a higher content of monoterpenes in SO4 which contradicts our findings as far as total monoterpenes are concerned since their APA was the highest in the most susceptible OIV class 1. Considering individual monoterpenes, a higher APA of *ß*-cyclocitral differentiated pure *V. vinifera* varieties (OIV classes 1, 3, and 5) from *V. riparia*, whereas linalool was significantly higher in OIV class 1 compared to OIV classes 3, 5, and 9. In contrast, these monoterpenes were detected in higher amounts in the leaves of resistant genotypes (i.e., BC4, Kober 5BB, SO4, and Solaris) compared to the susceptible Pinot noir [11]. Higher contents of linalool and neral were detected in Bianca and F12P60 suggesting their antimicrobial activity [15]. In addition to linalool, our research identified its volatile oxides, namely (*Z*)- and (*E*)-linalool oxides. Defence–related activity could be ascribed to (*Z*)-linalool oxide since its highest APA was detected in *V. riparia* (OIV 9) distinguishing this genotype from the others evaluated. Neral was detected in Solaris (OIV 7) with a high APA, but it was also found in the most susceptible varieties (OIV 1).

Aldehyde 4-pentenal distinguished *V. riparia* from other genotypes by its low APA, whereas Lazazzara et al., (2018) [11] detected a higher APA of (*E*)-2-pentenal in the resistant genotypes of BC4 and Kober 5BB compared to Pinot noir. As far as (*E,E*)-2,4-heptadienal and benzeneacetaldehyde are concerned, lower APAs were detected in resistant genotypes in both of these studies. The same authors found benzaldehyde to be more abundant in resistant varieties, whereas, in our study, it was most abundant in OIV classes 5 and 9, although a high APA was also detected in OIV class 1. Nevertheless, Chitarrini et al., (2017) [14] suggested benzaldehyde as a putative biomarker of resistance to *P. viticola* infection since it was detected in higher concentrations in infected samples of the resistant cultivar Bianca at 48 and 96 hpi. Bianca, Solaris, and F12P60, which possess at least one locus of resistance in their genomes, had a higher level of benzaldehyde [15]. Moreover, it was found that benzaldehyde acts as a promoter of salicylic acid (SA)–mediated defence, as it accumulated early and in high concentration in the plant metabolome with *Rpv12*–mediated resistance [38]. Salicylic acid acts as a phytohormone precursor of the volatile compound methyl salicylate, which is known for activating induced resistance upon attack by biotrophic microorganisms, such as *P. viticola* [12]. Aldehyde 2-hexenal was found in increasing APAs throughout the sampling terms and its high APAs were detected in OIV classes 1 and 5. In our experiment, however, the APA of nonanal decreased. Furthermore, the high APA of nonanal in OIV class 5, the same was detected in Solaris (OIV 7), which could be one of the possible commonalities related to lower susceptibility among these two OIV classes. Previously, a higher content of 2-hexenal was associated with the Solaris cultivar resistance, whereas a higher content of nonanal was detected in the leaves of the F12P127 genotype, and based on that, these VOCs were proposed as biomarkers of resistance [15].

Phenylethyl alcohol increased throughout the experiment and was detected in the highest APA in OIV class 9 corroborating the findings of Lazazzara et al., (2018) [11]. Throughout the experiment, the alcohols 1-hexanol and (*E*)-2-hexen-1-ol were found in ascending APAs, while their higher APAs in inoculated leaves distinguished them from non-inoculated leaves. OIV 5 varieties have the highest APA of (*E*)-2-hexen-1-ol, which suggests that it may play a role in the defence mechanism of *V. vinifera* varieties that are less susceptible to *P. viticola*. Both alcohols, together with 2-ethyl-1-hexanol and 1-octen-3-ol, have been identified as potential biomarkers of resistance in Bianca, Solaris, and F12P60 genotypes due to their higher concentration upon inoculation compared to Pinot noir, which is susceptible [15]. In our study, the APA of 2-ethyl-1-hexanol decreased with time and was higher in non-inoculated leaves and in varieties that are the most susceptible to *P. viticola* (OIV 1), whereas 1-octen-3-ol was not significant for any of these parameters.

Among esters, (*Z*)-3-hexenyl benzoate was proposed as a potential biomarker of resistance in previous research due to its higher up-regulation upon *P. viticola* inoculation in the resistant genotype of F12P60 compared to Pinot noir [15]. Similarly, we identified a relatively high APA of (*E*)-2-hexenyl benzoate in *V. riparia*. Methyl salicylate is synthesized from salicylic acid by salicylate methyl transferase and is widespread in plants as a volatile odorous compound associated with mint-like and green pepper aromas [39]. It was demonstrated that a higher concentration of methyl salicylate in stems, grapes, and consequently in red and white wines was related to vine diseases (downy mildew, grape black rot, Esca) suggesting a host plant-induced defence mechanism against fungal infection. Thus, methyl salicylate can serve as a volatile indicator of the vineyard’s infection status [40]. In our study, it was detected in the highest abundance in the resistant genotype of *V. riparia* as well as the highly resistant Solaris cultivar confirming its potent antifungal activity in these genotypes. Salicylic acid and methyl salicylate induce systemic acquired resistance and hypersensitive response (cell death) as a reaction to a pathogen attack [41]. Although methyl salicylate did not help distinguish the non-inoculated and inoculated samples, its higher APA was observed 48 h upon inoculation corroborating that the synthesis of this VOC is induced by a pathogen attack. Acting as a volatile form of a defence phytohormone, methyl salicylate systemically induces defence responses in plant parts and organs that are distant from the initial infection site. Moreover, these airborne signals can be perceived by uninfected neighbouring plants and induce a resistance reaction in them as well [12]. Previously, methyl salicylate has been proposed as a biomarker of downy mildew infection [42] and as a potential biomarker of resistance to *P. viticola* since it was detected in higher concentrations in Bianca compared to Pinot noir [15].

In previous studies that included field trials and in vitro leaf disc bioassay, three Croatian native grapevine varieties, namely Malvazija istarska, Ranfol, and Teran (OIV class 5), proved to be more resistant to *P. viticola* compared to other evaluated *V. vinifera* varieties [28]. Moreover, their constitutive polyphenolic profile of leaves, i.e., higher content of flavonol glycosides mostly, distinguish them from the more susceptible varieties [29]. Aiming to define VOCs that could be responsible for the defence mechanism of these three Croatian grapevine varieties, VOCs from OIV class 5 were compared with VOCs from OIV classes 7 and 9. Therefore, a few compounds with a higher APA upon inoculation in inoculated leaves compared to non-inoculated ones in OIV classes 5, 7, and 9 were observed, namely geranylacetone, *ß*-ocimene, and (*E*)-2-hexen-1-ol.

Geranylacetone belongs to the class of organic compounds known as acyclic monoterpenes. It is a component of essential oils in various plants including *Nelumbo nucifera* whose leaf extract has strong antioxidant properties [43]. Up to now, geranylacetone was not recognized as a biomarker of grapevine resistance to *P. viticola*, although it was detected in higher concentrations in the leaves of the resistant pyramided genotype F12P60 compared to Pinot noir [15]. Ricciardi et al., (2021) [8] did not find significant changes in the quantity of this compound upon inoculation in either Bianca or Mgaloblishvili. In this study, geranylacetone was found to have an ascending APA throughout the experiment and a significantly higher APA in each sampling term in the inoculated leaves of the native varieties Malvazija istarska and Ranfol, as well as the highly resistant Solaris cultivar, compared to non-inoculated leaves, indicating that geranylacetone may be involved in the defence mechanism of these cultivars.

Another possible indicator of resistance is *ß*-ocimene, a volatile organic compound that belongs to the class of monoterpenes. During T_2_ (48 hpi), its induced accumulation was observed in the inoculated leaves of the native variety Malvazija istarska, whereas it was never detected in the control leaves. *ß*-ocimene was detected in both inoculated and non-inoculated leaves of the *V. riparia* resistant genotype, although its APAs were higher in inoculated leaves in each term following inoculation. Previously, terpenes were often recognized as compounds associated with the defence mechanism against downy mildew [8,11,13]. Specifically, allo-ocimene was found to activate defence genes and induce resistance against *Botrytis cinerea* in *Arabidopsis thaliana* [44]. Functional properties of terpenes, such as farnesene, nerolidol, valencene, and ocimene were examined and found efficient in counteracting *P. viticola*. Not only were they synthesized in higher amounts in the resistant variety of Mgaloblishvili, but their antisporulant activity was also proved in ad hoc experimental inoculations in which disease severity and sporangia concentration were inhibited. Among these terpenes, ocimene was found to be the most effective [8].

As previously mentioned, the volatile alcohol (*E*)-2-hexen-1-ol was proposed as a biomarker of resistance in the study conducted by Ciubotaru et al., (2021) [15]. In our research, the same compound was detected in inoculated leaves of Teran and *V. riparia* in ascending APAs upon *P. viticola* inoculation. (*Z*)-3-hexenol, an alcohol with a similar structure, was detected in higher concentrations in the resistant *V. labrusca* and *V. riparia* genotypes compared to the susceptible *V. vinifera* varieties [45]. (*Z*)-3-hexenol is known to induce up-regulation of defence genes during the *Botrytis cinerea* infection of *Arabidopsis thaliana,* acting in the same way as allo-ocimene [44].

## 4. Materials and Methods

### 4.1. Preparation of samples

#### 4.1.1. Plant Material

The plant material needed for this experiment was prepared in the same way as in our previous research [29]. In short, plant material of 17 grapevine genotypes was used in this research. Out of these genotypes, 14 were Croatian native grapevine varieties. Chardonnay served as the susceptible control variety, while Solaris and *Vitis riparia* served as the partially resistant and resistant control genotypes, respectively (Table 1). Previously, they were distributed into the OIV classes of resistance by applying the leaf disc bioassay [28] according to the OIV 452-1 descriptor [Leaf: degree of resistance to *Plasmopara* (leaf disc test)] [46]. Therefore, each genotype was assigned to the relevant OIV class of resistance (Table 1 and Table 2). The leaf discs used for assigning each genotype to the appropriate OIV class of resistance were excised from the exact same leaves used for the analysis of volatile organic compounds. Moreover, the leaf discs were inoculated with the same *P. viticola* suspension as the leaves. The sporulation was evaluated on the seventh day upon inoculation.

In order to produce healthy leaves, hardwood cuttings were planted in regularly drop-irrigated pots under greenhouse conditions of 15 to 24 °C air temperature and 65 to 75% relative humidity during the cultivation period. The fourth and the fifth leaves beneath the apex were used since they are not mature enough to resist or tolerate the downy mildew disease with the exception of resistant genotypes. The leaves were transferred from the greenhouse to the laboratory, where they were rinsed with ultrapure water. There was no evidence of foliar diseases on the leaves at the time of sampling in the greenhouse.

#### 4.1.2. *Plasmopara viticola* Suspension Preparation

For the preparation of a dense and cloudy *P. viticola* suspension, naturally infected leaves were used that were taken from the vineyard where no chemical protection was applied. This suspension was applied to the abaxial leaf surfaces of the susceptible Chardonnay variety in order to produce fresh sporulation. The leaves were placed in in vitro laboratory conditions optimal for *P. viticola* propagation. The leaves with freshly developed sporulation were soaked in ultrapure water and the sporulation was removed using a soft brush until the water became cloudy. The suspension concentration was adjusted to 2 × 10^5^ spores mL^−1^ with a Neubauer cell counting chamber [47,48]. The freshly prepared suspension was used to inoculate the leaves of 17 genotypes.

#### 4.1.3. Inoculation and Incubation of the Leaves

Four leaves from each genotype were sampled and frozen at −20 °C until analysis (T_0_). The remaining 24 leaves per genotype were separated into two groups: mock-inoculated leaves (treated with ultrapure water) and leaves inoculated with *P. viticola* suspension. Each leaf was placed in a separate Petri dish (150 mm in diameter) on wet filter paper. The leaves were laid with the abaxial side up and sprayed with either ultrapure water or the *P. viticola* suspension. The Petri dishes were sealed with parafilm and placed in the climate chamber with optimal conditions for downy mildew development (air temperature of 20°C, air humidity of 80%). The samples were kept in dark for the first 24 h. Then, water or suspension droplets were removed with a sterile filter paper to prevent leaf decay. After that, a 16-h photoperiod was applied to simulate outdoor conditions [47,48]. At certain time points after inoculation [T_1_—24 h post-inoculation (hpi); T_2_—48 hpi; T_3_—96 hpi] [14,49,50], the samples were taken from the climate chamber and stored in the freezer (−20 °C) until freeze drying (lyophilization). For each genotype, additional leaves were inoculated beyond those required for volatile organic compound analysis to ensure that infection was successful.

### 4.2. Analysis of Volatile Organic Compounds 

#### 4.2.1. SPME-Arrow Extraction of VOCs

Before the analysis, the frozen leaves were lyophilized and ground into a powder using a MiniG Mill (SPEX Sample Prep, Metuchen, NJ, USA) (1 min, 1500 rpm). The SPME-Arrow extraction was carried out following the method described by Šikuten et al., (2021) [30]. In short, the SPME-Arrow extraction was conducted using the RSH TriPlus autosampler (Thermo Fisher Scientific Inc., Brookfield, WI, USA). A 100 mg sample was placed in 20 mL headspace screw-top vials with PTFE/silicone septum caps. 

The sorption conditions were as follows: the sample was incubated at 60 °C for 20 min, and then SPME-Arrow fiber DVB/CWR/PDMS (120 µm × 20 mm; Thermo Fisher Scientific Inc., Brookfield, WI, USA) was exposed for 49 min. Then, the fiber was inserted into the GC injector port operating in the splitless mode and desorbed at 250 °C for 10 min. All leaf samples were analysed in triplicate.

#### 4.2.2. GC–MS Analysis

The analytes were separated and detected using a TRACE^TM^ 1300 Gas Chromatographer coupled with an ISQ 7000 TriPlus quadrupole mass spectrometer (Thermo Fisher Scientific Inc., Bartlesville, OK, USA) equipped with a TG-WAXMS A capillary column (60 m × 0.25 mm × 0.25 µm film thickness; Thermo Fisher Scientific Inc., Bartlesville, OK, USA). The volatile compounds injected into the inlet were delivered to the column in the splitless mode. Helium was used as a carrier gas at a constant flow rate of 1 mL/min. The oven temperature program was as follows: an initial temperature of 40 °C was maintained for 5 min, increased by 2 °C every minute until the temperature reached 210 °C, and then it was maintained for 10 min. The MS spectra were recorded in the electron impact ionization mode (EI) with an ionization energy of 70 eV. The mass spectrometry was performed in the full scan mode between 30 and 300 *m*/*z*. Chromeleon^TM^ Data System was utilised to process the obtained data (Thermo Fisher Scientific Inc., Bartlesville, OK, USA). The volatile compounds were identified by comparing the recorded mass spectrum to the information contained in the Wiley Registry 12th Edition/NIST Spectral Library. The Retention Index (RI) was calculated using alkane standards C_8_-C_20_ (Sigma Aldrich, St. Louis, MO, USA) in accordance with the equation in Song et al., (2019) [51] and compared to previously published results [52,53]. The retention indices are presented in Table S1. All results are expressed as absolute peak areas (APA).

### 4.3. Statistical Analysis

Factorial ANOVA was performed on the absolute peak area (APA) of the volatile organic compounds to define the effects of treatment (non-inoculated vs. inoculated samples), the classes of resistance, and the terms of sampling after inoculation. The differences between the means of specific factors were evaluated by Duncan’s multiple range test at a confidence level of 95% (*p* < 0.05). Since there was no treatment in T_0_ (the sampling period preceding inoculation), it was excluded from the factorial ANOVA used to define the exact effect of each individual factor (Table S3).

To compare the differences in VOCs response between inoculated and non-inoculated leaves, correlations were calculated between VOCs APA and the sampling time from T_0_ to T_3_ (time in days after the T_0_) separately for each genotype and treatment (Table S4) using Pearson’s coefficient. Principal component analysis (PCA) was performed using the average values of APA of different VOC groups for treatment (non-inoculated and inoculated), which were sampled in different terms before and upon inoculation (0, 24, 48, and 96 hpi) belonging to different OIV classes of resistance. The XLSTAT statistical and data analysis solution (Addinsoft, 2021, New York, NY, USA) was used for statistical analyses.

## 5. Conclusions

This work provides an insight into the profiles of leaf volatile organic compounds (VOCs) before and after *P. viticola* inoculation in 15 *V. vinifera* varieties, 14 of which are native to Croatia, as well as the resistant Solaris and *V. riparia* genotypes. Sesquiterpenes proved to be the most appropriate for distinguishing highly resistant genotypes (OIV classes 7 and 9) from *V. vinifera* varieties (OIV classes 1, 3, and 5), as well as non-inoculated from inoculated leaves. Moreover, their ascending APA throughout the experiment was more pronounced in inoculated samples confirming that the synthesis of sesquiterpenes was upregulated by the *P. viticola* infection. However, neither sesquiterpenes nor other groups of VOCs separated the OIV resistance classes into different groups. Nevertheless, a few compounds, namely geranylacetone, *ß*-ocimene, and (*E*)-2-hexen-1-ol were identified in the Croatian native grapevine varieties of lower susceptibility to *P. viticola* (Malvazija istarska, Ranfol, and Teran) that could be involved in their defence mechanism since the same was detected in Solaris or *V. riparia*. However, due to the fact that plant volatilomics is highly dependent on numerous stressors, additional research is necessary to determine the direct role of these VOCs by conducting experiments on non-detached, in vivo leaves.

## Figures and Tables

**Table 1:** Genotypes, additional information on the plant material, and genotypes’ corresponding OIV classes of resistance to *P. viticola* (OIV 452-1) according to OIV [46]. Genotypes in bold were used as controls.

Genotype (Accession Name)	Holding Institute	Material Source ID (EURISCO)	VIVC Code	Species	OIV 452-1
Belina starohrvatska	HRV041	VIT00233	5374	*Vitis vinifera* subsp. *vinifera*	1
Debit	HRV041	VIT00017	10423	*Vitis vinifera* subsp. *vinifera*	1
Grk	HRV041	VIT00030	5066	*Vitis vinifera* subsp. *vinifera*	1
Moslavac	HRV041	VIT00052	4292	*Vitis vinifera* subsp. *vinifera*	1
Plavac mali	HRV041	VIT00060	9549	*Vitis vinifera* subsp. *vinifera*	1
Babić	HRV041	VIT0002	844	*Vitis vinifera* subsp. *vinifera*	3
**Chardonnay**	HRV041	CL-277*	2455	*Vitis vinifera* subsp. *vinifera*	3
Kraljevina	HRV041	VIT00035	24904	*Vitis vinifera* subsp. *vinifera*	3
Plavina	HRV041	VIT00062	9557	*Vitis vinifera* subsp. *vinifera*	3
Pošip	HRV041	VIT00065	16018	*Vitis vinifera* subsp. *vinifera*	3
Škrlet	HRV041	VIT00085	22983	*Vitis vinifera* subsp. *vinifera*	3
Tribidrag	HRV041	VIT00013	9703	*Vitis vinifera* subsp. *vinifera*	3
Malvazija istarska	HRV041	VIT00047	7269	*Vitis vinifera* subsp. *vinifera*	5
Ranfol	HRV041	VIT00070	9908	*Vitis vinifera* subsp. *vinifera*	5
Teran	HRV041	VIT00087	12374	*Vitis vinifera* subsp. *vinifera*	5
**Solaris**	DEU455	20340 *	20340	*Vitis vinifera* subsp. *vinifera*	7
***Vitis riparia***	DEU098	4609 *	4609	*Vitis riparia*	9

Plant material from the vineyard on Experimental station Jazbina, University of Zagreb, Faculty of Agriculture, Department of Viticulture and Enology, Cv. Chardonnay, clone CL-277. * According to VIVC. VIVC—Vitis International Variety Catalogue (https://www.vivc.de, the access date: 15 November 2022). OIV 452-1—Descriptor for leaf: degree of resistance to *Plasmopara* (leaf disc test).

**Table 2:** Phenotypes of representative inoculated leaf discs at the time of the *Plasmopara viticola* sporulation evaluation.

Representative leaf disc	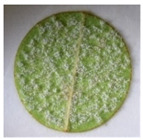	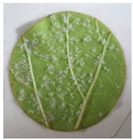	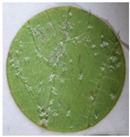	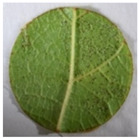	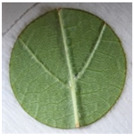
Genotype	Plavac mali	Babić	Malvazija istarska	Solaris	*Vitis riparia*
OIV resistance class	1	3	5	7	9
Surface covered with sporulation (%)	61–100	41–60	21–40	1–20	0

## Data Availability

The data presented in this study are available on request from the corresponding author.

## Supplementary Materials

The following supporting information can be downloaded at: https://www.mdpi.com/article/10.3390/plants12020404/s1, Table S1: Retention indices, Table S2: ANOVA Interactions, Table S3: ANOVA_Term_Treatment_OIV class, Table S4: Correlations.
